# The Effectiveness of Saline Load Test in Detecting Simulated Traumatic Elbow Arthrotomies: A Cadaveric Investigation

**DOI:** 10.7759/cureus.20793

**Published:** 2021-12-29

**Authors:** Alexandra C Ferre, Ahmed K Emara, Maria A Maurant, Andrew N Steckler, Brandon Merryman, Jessica L Churchill

**Affiliations:** 1 Department of Surgery, Cleveland Clinic, Cleveland, USA; 2 Department of Orthopedic Surgery, Cleveland Clinic, Cleveland, USA

**Keywords:** infection, cadaveric study, arthrotomy, saline load, elbow trauma

## Abstract

Background

The saline load test has not been well explored in the elbow. We aimed to determine 1) the saline infusion volume needed for 90%, 95%, and 99% sensitivity in detecting elbow arthrotomy; and 2) factors associated with higher volume at detection using sixteen forequarter upper extremity amputation cadavers.

Methods

Sixteen fresh-frozen forequarter upper extremity amputations were procured, and demographic data, including age, body mass index (BMI), and laterality, were recorded. The olecranon process, radial head, and the lateral epicondyle were palpated, and elbow arthrotomy was consistently performed at the direct lateral arthroscopic portal site. The elbow joint was loaded with saline mixed with methylene blue (concentration: 2 mg/300 mL) using an 18-gauge needle inserted just medial to the triceps tendon 2 cm superior to the olecranon.

Results

Mean volume for extravasation was 12.2 mL ±6.26. Volume needed for 90%, 95%, and 99% sensitivities were 21 mL, 23 mL, and 25.4 mL. Linear regression demonstrated that increasing age was associated with lower volume to extravasation (OR: 0.67; 95% CI: 0.48-0.932; p=0.037), while BMI (p=0.571) and extremity laterality (p=0.747) did not affect the volume.

Conclusions

The saline load test can be effective in diagnosing the violation of the elbow joint in traumatic injuries. This test should be used in conjunction with the clinical examination and radiographs before operative decisions are made. We recommend using ≥26 mL to rule out traumatic elbow arthrotomy.

## Introduction

Elbow injuries represent over 10.5% of upper extremity-related department visits, of which 7% are attributable to periarticular lacerations [1]. Various mechanisms may be implicated in such injuries, including motor vehicle accidents, falls from height, as well as stab and gunshot wounds [2]. While joint penetration may be evident with large wounds or open fractures, determining joint involvement with smaller lacerations solely through inspection is unreliable [3].

The presence of concurrent elbow arthrotomy may necessitate urgent irrigation with/without necrotic tissue debridement to avoid the development of septic arthritis, a devastating complication with associated irreversible joint destruction in as little as three days [4,5]. Notably, such aggressive interventions would not be warranted in the absence of joint arthrotomy. As such, accurate detection of joint capsule penetration on presentation is crucial to facilitate appropriate and timely downstream management [6,7]. Published investigations have demonstrated poor accuracy of computerized tomography (CT) scans in diagnosing traumatic elbow arthrotomy. Kupchick et al. [8] recently reported that zero out of ten iatrogenically arthrotomized cadaveric elbows demonstrated evidence of intra-articular air on CT, despite repetitive ranging of the joint. In the setting of possible traumatic arthrotomy, the saline load test (SLT) is the most common and accepted diagnostic modality [6]. Therefore, in addition to being universally available, the SLT adds value compared to advanced imaging alternatives, which demonstrate lower sensitivities yet greater costs, thereby proving crucial [8].

SLT consists of an injection of a certain volume of sterile saline into a given joint separate from the site of injury, whereby extravasation of saline from the injury site is a positive result that indicates a capsular breach [6,7]. Identifying the sensitivity of detecting arthrotomy through extravasation per injected saline volume is critical since underloading the joint may potentially lead to missed capsular violations. Conversely, overloading the joint with saline exposes patients to unnecessary discomfort due to increased intracapsular pressure and/or repetitive injection, in addition to the risk of intraarticular bacterial inoculation. SLT has been studied in the knee and ankle, with the recommended infusion volumes reported as 155 to 194 mL and 10 to 60 mL, respectively [9-16]. However, there is substantial controversy regarding the fluid volume required for elbow injection [3,4]. While some reports describe a 100% sensitivity at 20ml, others demonstrated that 95% sensitivity was only attained at 40ml of injected volume [3,8]. Such discrepancy is attributable, in part, to extraarticular needle placement and variation in the size of arthrotomy. Furthermore, the use of a surgical scalpel creates a homogenous clean-edged surgical incision-like arthrotomy that may not simulate penetrating lacerations.

Therefore, the purpose of this study was to evaluate; 1) the amount of fluid needed and 2) the subsequent sensitivity of the SLT per injected volume in identifying intra-articular arthrotomies of the elbow using a reproducible technique that simulates common elbow injuries. Furthermore, we aimed to characterize the potential association between specimen body mass index (BMI), age and laterality, and the volume required for extravasation.

## Materials and methods

Study design

After institutional approval was obtained, sixteen fresh-frozen forequarter upper extremity amputations were procured, and demographic data, including age, body mass index (BMI), and laterality, were recorded. All specimens were thawed, followed by a thorough examination of the range of motion and for any evidence of previous elbow, distal upper arm, and proximal forearm trauma or surgery. All diagnostic and interventional procedures were performed by a senior orthopedic surgery resident supervised by a professor of human anatomy. The current study was deemed exempt from Institutional Review Board Approval due to the use of procured de-identified cadaveric samples that are not associated with identifiable patients.

Arthrotomy technique

The Olecranon process, radial head, and the lateral epicondyle were palpated, and elbow arthrotomy was consistently performed at the direct lateral arthroscopic portal site (the “soft spot”). This site was chosen due to its minimal soft tissue protection, making it susceptible to penetrating injuries, in addition to being a reliable site that ensures capsular breach. Arthrotomy was performed using a perpendicularly directed 4.5 mm trochar without prior skin incision with a scalpel in an attempt to simulate penetrating traumatic injuries of the elbow closely. The intra-articular location of the arthrotomy was confirmed through trapping the trochar in the ulnohumeral joint.

Saline loading

Following arthrotomy, the elbows were ranged to avoid potential soft tissue plane alignment and the subsequently easier extravasation. The elbow joint was then loaded with saline mixed with methylene blue (concentration: 2 mg/300 mL) using an 18-gauge needle inserted just medial to the triceps tendon 2 cm superior to the olecranon with the elbow in 90 degrees of flexion. During the injection, the known arthrotomy site was observed for leakage. If no leakage occurred after loading 10 mL of fluid, the elbow was taken through a full range of motion two times. If still no leakage was appreciated at the arthrotomy site, the elbow was again infused with fluid in 2 mL increments until outflow. All injections were confirmed as intra-articular by demonstrating methylene blue staining of the joint through post-experimentation open exploration.

Outcome measures and statistical analyses

The primary outcome of the current study was the volume of intra-articular saline injection required to produce visible extravasation at the site of elbow arthrotomy. As such, the injected volume at extravasation was recorded for each specimen. Descriptive statistics, including mean ± standard deviation, range, medians, and percentiles, were computed the fluid volume required to achieve extravasation [17,18]. The aforementioned parameters were utilized to estimate the required volumes for 90%, 95%, and 99% diagnostic sensitivities. Furthermore, a linear regression model was constructed to evaluate potential confounding effects of cadaver age, body mass index, and laterality on the volume required for extravasation. All statistical analyses were implemented in R v.3.5.0 (R Foundation for Statistical Computing, Vienna, Austria), and statistical significance was set at an alpha level of 0.05 (p<0.05).

## Results

**Figure 1:**
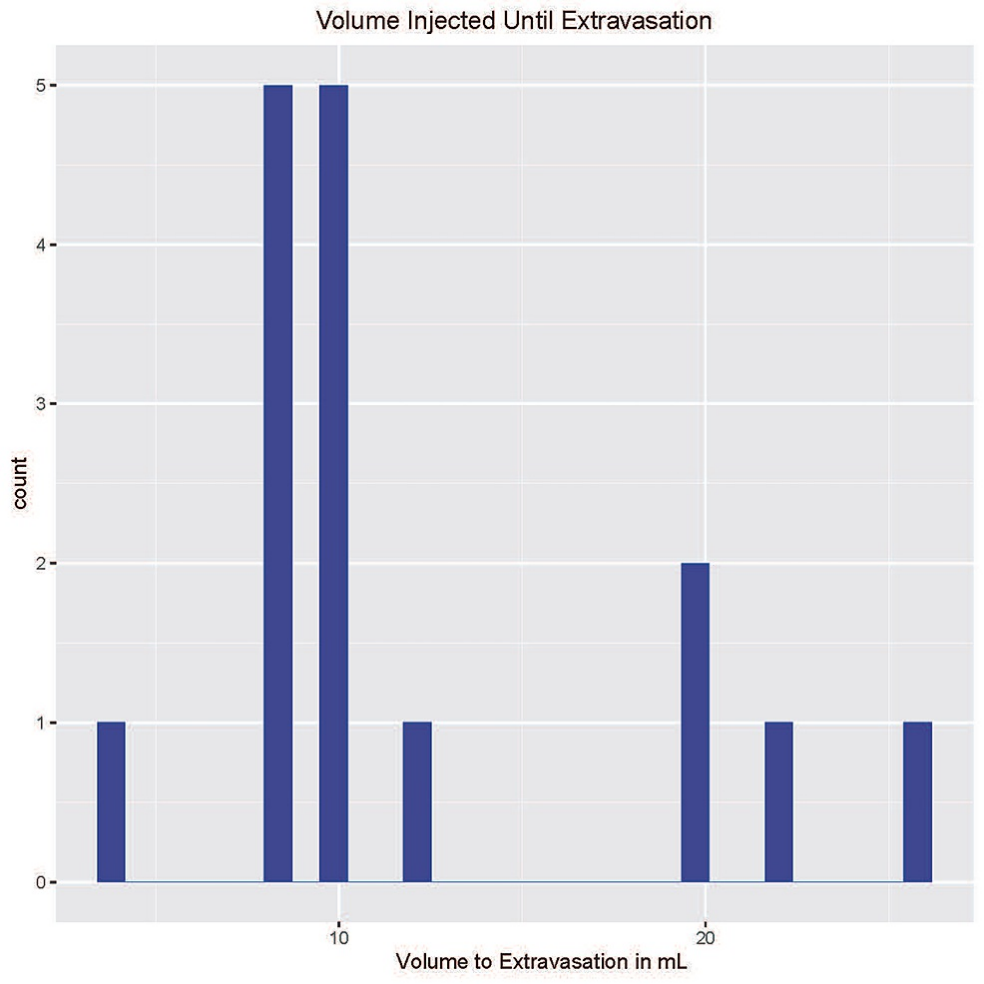
Histogram demonstrating the number of cadaveric specimens at each saline volume required to achieve extravasation

Careful examination revealed that all specimens were free of evidence of previous trauma or surgery. The cadaveric specimens' mean age was 76.1 years ± 10.04, and the mean BMI was 22.94 kg/m2 ± 4.61. The saline volume required for extravasation ranged from 4 ml up to 26 ml (Figure 1). The mean and median volumes required to achieve extravasation were 12.2 ml ± 6.26 and 10 ml, respectively.

Using the aforementioned protocol of intraarticular saline injection, a total of 23 ml was required to attain a 95% diagnostic sensitivity for the presence of elbow arthrotomy. Similarly, 21 ml and 25.4 ml were needed to detect elbow arthrotomies at 90% and 99% sensitivities, respectively. Linear regression demonstrated that increasing age was associated with lower volume to extravasation (OR: 0.67; 95% CI: 0.48-0.932; p=0.037), while BMI (OR: 0.57; 95CI: 0.64-2.194; p=0.571) did not significantly influence the required saline load. Similarly, extremity laterality did not significantly affect extravasation volume where sided extremities had similar odds (OR: 2.47; 95% CI: 0.012-528.7; p=0.747) of extravasating at a certain volume compared to left-sided ones.

## Discussion

Periarticular lacerations with possible joint penetration are common traumatic injuries. While the knee is the most frequently involved joint, the elbow is the second most common [3,19]. Open elbow injuries have the potential to cause significant morbidity if not identified and treated promptly [6]. The present study found that utilizing a saline volume of 26 ml coupled with intermittent ranging at 10 ml intervals can effectively detect 99% of elbow arthrotomies within a cadaveric sample. Furthermore, we found that the required volume to extravasation may be reduced among specimens with greater age.

The saline load test for open arthrotomy has been well studied in various joints such as the knee and ankle, but there is a paucity of literature for the elbow joint. Feathers et al. [3] utilized 36 cadaveric specimens to investigate the saline volume required for extravasation through a direct posterior arthrotomy. The authors reported that a total of 40 ml was required to identify arthrotomies at 95% sensitivity. Such load is markedly greater than that described by Kupchick et al. [8], who reported 100% sensitivity of detecting elbow arthrotomies using 20 ml of saline. Of note, the posterocentral arthrotomy utilized by Feather et al. [3] was implemented through a longitudinal split of the triceps tendon using a scalpel. The closely approximated ends may exert a valve-like mechanism that increases the pressure required for extravasation and subsequently raise the needed saline volume. The present study found a 99% sensitivity of arthrotomy detection at 26 ml. This was achieved using the direct lateral arthroscopic portal site (the "soft spot") that affords lower soft tissue resistance. Furthermore, all soft tissue layers were penetrated solely using a trochar in an attempt to provide a closer simulation of penetrating injuries than that attained through a skin incision followed by a trochar-mediated arthrotomy.

While arthrotomy location variation is an acknowledged confounder, the association between specimen characteristics and saline load volume has not been previously described [3,13,14]. The present investigation was the first to highlight a significant association between greater age at specimen collection and diminished SLT required for extravasation [20]. Conversely, there was no significant association between specimen BMI or laterality and intraarticular saline volume required for extravasation. However, sample size limitations preclude a definite assertion of lack of association.

Saline loading was performed with elbows at 80-90 degrees of flexion. Literature indicates that the highest intraarticular elbow capacity - and subsequently lowest pressures - is attained around 80 degrees of elbow flexion [21,22]. This suggests that the current model accounts for intraarticular space at its highest capacitance and that extravasation may be attained at lower extravasation volumes if elbows are tested at higher or lower degrees of extension or flexion, respectively.

This study had several limitations. First, we used cadaveric elbows, which do not precisely replicate the live elbows and joint tissue on which clinical saline load tests are performed. Moreover, the cadaveric elbows overly represent older, presumably less healthy specimens than the typical trauma patient's elbow [23]. The cadaveric elbows' tissue may be less compliant, and the typical traumatic joint effusion is not present in the cadaveric elbows. Second, we only used one arthrotomy site. As it would be impossible to represent all potential elbow injury patterns. However, the lateral arthrotomy site is an appropriate model for a common traumatic injury. Finally, sample size limitations may preclude the detection of significant associations between cadaver BMI or laterality and saline volume required for arthrotomy detection.

## Conclusions

In conclusion, this study shows that the saline load test can be effective in diagnosing the violation of the elbow joint in traumatic injuries. It is not an absolutely definitive test and should be used in conjunction with the clinical examination and radiographs before operative decisions are made. Furthermore, we found the saline load test's sensitivity can be increased with the addition of joint ranging and the injection of higher fluid volumes, with a point of diminishing returns in sensitivity at 26 ml load. Further investigations are required to assess the impact of patient demographics, BMI, and co-morbidities on the sensitivity of the SLT.
